# Idiopathic subglottic stenosis with an inguinal hernia in children: A case report

**DOI:** 10.1097/MD.0000000000036086

**Published:** 2023-11-17

**Authors:** Sai Liang, Ji Wang, Kai Song, Ming Yu, Zhengpeng Gong

**Affiliations:** a Department of Clinical Medicine, Guizhou Medical University, Guiyang City, Guizhou Province, China; b Department of Otorhinolaryngology, The Affiliated Hospital of Guizhou Medical University, Guiyang City, Guizhou Province, China.

**Keywords:** children, idiopathic subglottic stenosis (iSGS), inguinal hernia (IH), pediatric inguinal hernia (PIH)

## Abstract

**Rationale:**

Idiopathic subglottic stenosis is a fibrotic condition of unknown origin that results in blockage of the central airway in the subglottic region. It is widely acknowledged that subglottic stenosis is a relatively uncommon structural anomaly that is difficult to operate on and cure due to its anatomical location. Inguinal hernias are well-established to be prevalent in infants and youngsters. We present a case of subglottic stenosis in a child complicated with an inguinal hernia (IH).

**Patient concerns:**

A 7-year-old female was admitted to our hospital with a 1-month history of progressive bulging in the left lower quadrant of the abdomen. She complained of no stomach discomfort, distension, or dyspnea, but her family reports that the patient usually wheezes during moderate exertion and has no family history of asthma or lung illness. However, for unclear reasons, the infant experienced shortness of breath following training. A chest CT scan was unremarkable. Below the glottis, a membranous stenosis was discovered. The stenosis beneath the glottis was discovered using dynamic laryngoscopy.

**Diagnoses:**

Idiopathic subglottic stenosis with an IH.

**Interventions:**

An otorhinolaryngologist employed a carbon dioxide laser to eliminate the subglottic stenosis. Following successful intubation by the anesthesiologist, pediatric surgeons performed laparoscopic high ligation of the hernial sac.

**Outcomes:**

After 1 month, a repeat laryngoscopy revealed significant expansion of the subglottic stenosis, accounting for the improvement in respiratory symptoms.

**Lessons:**

The present case raises awareness that surgeons should be more vigilant about respiratory complications in patients with an IH. Early diagnosis and treatment of respiratory illnesses are critical for patients undergoing endotracheal intubation.

## 1. Introduction

Subglottic stenosis (SGS) is a condition in which the central airway is obstructed at the level between the glottis and the first 2 tracheal cartilage rings.^[1]^ After a comprehensive investigation, an idiopathic subglottic stenosis (iSGS) diagnosis is established when no obvious reason is identified. It is well-recognized that iSGS is an uncommon and potentially fatal condition characterized by extrathoracic blockage of the subglottic and proximal trachea.^[2]^ affecting around 1 in 400,000 women between 30 and 50.^[1,3]^ Due to the uncommon occurrence and presentation of iSGS in healthy persons, early diagnosis is often difficult, resulting in treatment delays.^[4,5]^ The subglottic area originates underneath the vocal cords and extends to the cricoid cartilage’s lower margin, composed of the thyroid cartilage’s lower margin, the cricothyroid cell membrane, the cricoid cartilage, and the surrounding soft tissue. The subglottic airway segment is widely acknowledged as the narrowest section of the airway in newborns and young children. SGS is characterized by a lumen diameter of 4mm in full-term babies and 3 mm in preterm newborns.^[6]^

Numerous hypotheses have been advanced regarding the disease’s etiology; however, no consensus has been reached about the optimal approach for therapy. Patients may gradually exhibit symptoms during childhood and development, depending on whether idiopathic subglottic stenosis develops at an early stage, progresses with age, or both. The most common symptoms are progressive dyspnea, hoarseness, and coughing. This condition is associated with diagnostic delays and is frequently misdiagnosed as asthma. The detection of stenosis during endoscopic examination is essential to establish the diagnosis.^[3,7,8]^ A wide range of therapeutic modalities is available for this patient population, including periodic dilation, circumferential tracheotomy, and tracheotomy.^[9]^ Endoscopic balloon dilation is the most commonly used procedure, followed by radial incision with carbon dioxide laser.^[10]^

Inguinal hernia (IH) is one of the most common surgically treated diseases in children.^[11]^ and herniorrhaphy remains the mainstay of treatment.^[12]^ The reported incidence in children is 0.8% to 4.4%.^[13]^ and the most common etiology is a patent processus vaginalis. In such cases, high ligation of the hernial sac is associated with excellent results.^[14]^

Herein, we sought to share our experience in surgically treating inguinal hernias (IH) with subglottic stenosis. This case improves current knowledge of IH associated with airway stenosis to raise the awareness of clinicians for early discovery and therapy of subglottic stenosis.

## 2. Case presentation

**Figure 1.**
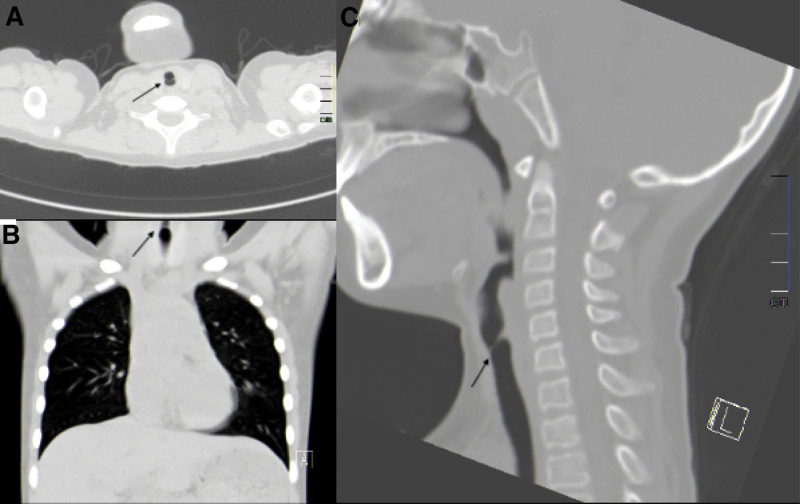
Axial (A), coronal (B), and sagittal (C) CT scan of the trachea depiction subglottic stenosis.

**Figure 2.**
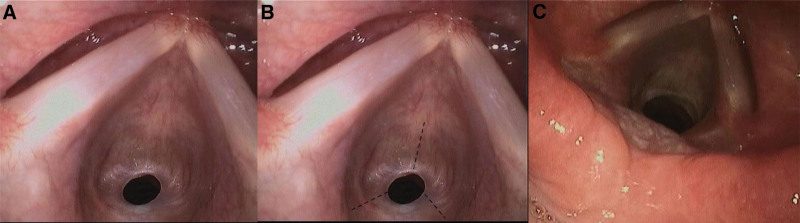
Membranous stenosis under glottis (A); radial incisions on the concentric scar (B); 1 month after the operation, the electronic laryngoscope showed that the stenosis became spacious (C).

A 7-year-old female was admitted to our hospital with a 1-month history of progressive bulging in the left lower quadrant of the abdomen. She complained of no stomach discomfort, distension, or dyspnea, but her family reports that the patient usually wheezes during moderate exertion and has no family history of asthma or lung illness. However, for unclear reasons, the infant experienced shortness of breath following training. A chest CT scan was unremarkable. Below the glottis, a membranous stenosis was discovered (Fig. 1). The stenosis beneath the glottis was discovered using dynamic laryngoscopy (Fig. 2A). An experienced multidisciplinary team of different specialties was set up, including anesthesia, otorhinolaryngology, pediatric surgery, and pediatric critical care. The multidisciplinary team concluded that the optimal management was concurrent ligation of the IH sac with intubation for subglottic stenosis. Tracheotomy is usually indicated if the blood oxygen saturation is decreased and airway blockage occurs during surgery. However, extubation may be difficult in children following tracheotomy, and subglottic stenosis may be exacerbated. Accordingly, tracheotomy should be avoided while treating subglottic stenosis. Furthermore, in such cases, intubation must be conducted in the pediatric critical care unit for ventilator support.

**Figure 3.**
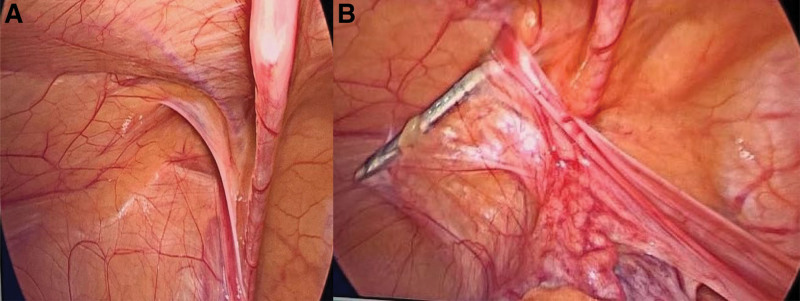
Laparoscopic observation of repair (A and B).

Thus, after consulting the patient’s family and receiving their consent, an otorhinolaryngologist employed a carbon dioxide laser to eliminate the subglottic stenosis. Circular stenosis was discovered around 0.5 cm under the glottis during the procedure. A CO_2_ laser was used to cauterize the tracheal mucosa and make radial incisions on the concentric scar (Fig. 2B). The stricture progressively transformed into a hypertrophic scar with contracture formation. Following successful intubation by the anesthesiologist, pediatric surgeons performed laparoscopic high ligation of the hernial sac. Figure 3 depicts the intraoperative scenario (Fig. 3). Following surgery, the youngster was sent to the pediatric critical care unit and placed on ventilator support. After 1 month, a repeat laryngoscopy revealed significant expansion of the subglottic stenosis, accounting for the improvement in respiratory symptoms (Fig. 2C).

## 3. Discussion

It is well-established that iSGS is the third most common congenital laryngeal anomaly behind laryngomalacia and vocal cord paralysis. Nonetheless, it is the most common laryngeal anomaly in infants under the age of one receiving tracheotomy.^[15]^ In our case, the patient had no prior history of endotracheal intubation, and the cause of the tracheal stenosis was unclear. Inguinal hernia is a clinical condition resulting from the inadequate or delayed fusion of the processus vaginalis, warranting surgery.^[16]^ In recent years, much emphasis has been placed on determining the underlying mechanism of IH disease development through gene expression investigations. It has been established that the decline in fascial tension resistance and elasticity of the transversus abdominis with age is morphologically determined. These deficiencies may contribute to the increased prevalence of hernia.^[16,17]^ In this situation, the IH may have been induced by increased activity as a result of growing up or by a progressive deterioration of tracheal stenosis, resulting in greater abdominal breathing and a chronic increase in abdominal pressure. Thus, our case reminds pediatric surgeons to be circumspect about IH patients presenting with respiratory symptoms. An IH can also occur due to increased abdominal pressure induced by persistent dyspnea. Additionally, respiratory illnesses can significantly increase the likelihood of requiring general anesthesia. Given that the subglottic tracheal stenosis might make it challenging to establish a patent airway, it is essential to conduct a preoperative respiratory assessment.

A chest X-ray has limited utility in diagnosing iSGS, while CT scans of the chest and neck with three-dimensional reconstruction give critical information for diagnosing and treating iSGS.^[18]^ Additionally, endoscopic assessment (laryngoscope or bronchoscope) is the gold standard for diagnosing and establishing treatment regimens for iSGS.^[1]^ Endoscopy enables the determination of the location, scope, and complexity of iSGS. Concentric reticular stenosis <1 cm in length is termed “simple,” whereas stenosis with structural abnormalities is “complex.”^[1,15]^In this instance, the stenosis was referred to as “simple” stenosis. Endoscopic treatment may be considered for patients with “simple” concentric reticular stenosis that does not affect the cartilage, whereas complicated lesions require surgical repair.

Endoscopy can be used as the primary or adjuvant treatment before and after airway reconstruction. Endoscopy is indeed a relatively less invasive technique. Subglottic stenosis can be treated by radiating incisions in concentric scars using a laser beam, a cautery knife, or other methods.^[1,19]^

## 4. Conclusion

Notwithstanding that subglottic stenosis and IH have been vastly documented in the literature, there have been no reports of concurrent SGS and IH. Herein, we documented a case of IH secondary to increased abdominal pressure caused by long-term subglottic stenosis. Clinically, subglottic stenosis is sometimes difficult to identify early and is frequently misdiagnosed as asthma or other lung illnesses. Importantly, the present case raises awareness that surgeons should be more vigilant about respiratory complications in patients with an IH. Early diagnosis and treatment of respiratory illnesses are critical for patients undergoing endotracheal intubation.

## Acknowledgments

The authors would like to thank the patient and her family for providing informed consent for publication.

## Author contributions

**Conceptualization:** Sai Liang, Kai Song, Ming Yu, Zhengpeng Gong.

**Data curation:** Sai Liang, Ji Wang, Kai Song, Ming Yu, Zhengpeng Gong.

**Formal analysis:** Ji Wang, Kai Song, Ming Yu, Zhengpeng Gong.

**Funding acquisition:** Ming Yu.

**Investigation:** Ji Wang, Zhengpeng Gong.

**Methodology:** Sai Liang, Ji Wang, Kai Song, Ming Yu, Zhengpeng Gong.

**Project administration:** Sai Liang, Zhengpeng Gong.

**Resources:** Sai Liang, Kai Song, Ming Yu, Zhengpeng Gong.

**Software:** Sai Liang, Kai Song, Ming Yu, Zhengpeng Gong.

**Supervision:** Sai Liang, Ji Wang, Kai Song, Ming Yu, Zhengpeng Gong.

**Validation:** Sai Liang, Ji Wang, Zhengpeng Gong.

**Visualization:** Sai Liang, Ji Wang, Ming Yu, Zhengpeng Gong.

**Writing – original draft:** Sai Liang, Ji Wang.

**Writing – review & editing:** Sai Liang, Ji Wang, Ming Yu, Zhengpeng Gong.
